# Adding employment status in the medical record demonstrates its importance as a social determinant of health

**DOI:** 10.1093/jamiaopen/ooaf108

**Published:** 2025-10-01

**Authors:** Laura E Breeher, Samantha Westphal, Tammy Green, Alzhraa Abbas, Clayton T Cowl

**Affiliations:** Division of Public Health, Infectious Disease, and Occupational Medicine, Mayo Clinic, Rochester, MN 55902, United States; Occupational Health Services, Practice Administration, Mayo Clinic, Rochester, MN 55902, United States; Division of Public Health, Infectious Disease, and Occupational Medicine, Mayo Clinic, Rochester, MN 55902, United States; Division of Public Health, Infectious Disease, and Occupational Medicine, Mayo Clinic, Rochester, MN 55902, United States; Division of Public Health, Infectious Disease, and Occupational Medicine, Mayo Clinic, Rochester, MN 55902, United States; Division of Public Health, Infectious Disease, and Occupational Medicine, Mayo Clinic, Rochester, MN 55902, United States; Division of Pulmonary and Critical Care Medicine, Mayo Clinic, Rochester, MN 55902, United States

**Keywords:** social determinants of health, employment, workforce retention, healthcare

## Abstract

**Objective:**

To share findings of a quality improvement initiative to capture employment status in the electronic medical record (EMR) to mitigate the potential impact of work loss on the health of patients by utilizing the results to identify eligible individuals for resources to assist return-to-work efforts.

**Materials and Methods:**

Patients self-identified employment status through a structured new social determinants of health (SDOH) question within the EMR. An electronic outreach campaign was developed to provide information via the patient portal detailing services within healthcare and the community that could benefit the patients.

**Results:**

Over the course of 12 months, 2059 patients were identified from the employment SDOH question. Resources to support stay at work and return to work efforts were provided to patients through an automated electronic portal campaign with 87% of patients reading the message and 7% engaging with a healthcare return to work case manager.

**Discussion:**

Loss of employment has detrimental impacts on individual and population health. Most EMRs do not capture information on employment status. Adding this simple question identified individuals with potential gaps in SDOH, and in this case allowed specific resources to be shared with patients with an illness or injury that was acutely impacting work.

**Conclusion:**

Medical center decision makers and EMR programmers should consider adding employment status as a social determinant of health.

## Background and significance

The importance of factors such as sleep, nutrition, social connections, and stress affecting the personal health of patients is well accepted.^1–4^ Employment status and the ability to work are crucial to financial stability and access to healthcare insurance for many individuals. The importance of work status is demonstrated by employment being included as a domain within the SDOH concept of economic stability framework for health equity shared by the World Health Organization.^5^ However, the positive impact of employment on health and, conversely, the harmful effects of unemployment, are often overlooked.^6^^,^^7^ In a large study of a prospective cohort of over 100 000 individuals, low socioeconomic status was associated with increased risk of developing 32% (18 of 56) medical conditions.^8^ Chronic health conditions such as mental health disorders can have fluctuating impacts on employment due to exacerbations of illness. During the COVID-19 pandemic, the important connection between health and work ability were highlighted.^9^ Although many individuals with chronic disease who were at high risk for severe illness due to viral spread stayed home or requested special temporary accommodations at work, millions of these workers lost their jobs altogether.^10^^,^^11^ This left in gaps in the workforce and called attention to the lack of resources within healthcare to identify patients whose medical condition(s) were impacting work.

In the past, employment status has not been routinely captured in electronic medical records (EMRs). When included, employment information is typically limited to the employer’s name and is often linked to medical insurance status. Since work is a dynamic variable that may change over time and is not systematically captured in the healthcare EMR, the limited information present is often inaccurate and not useful. One example of the impact from absence of electronic tools in the EMR focused on employment status was the obstacle to identification of patients who would benefit from services through the Minnesota Retaining Employment and Talent After Injury and Illness Network (MN RETAIN),^12^ an IRB approved program involving a randomized controlled trial examining embedding a return-to-work case manager into the healthcare team in an effort to provide support for injured or ill individuals attempting to maintain work or return to work. Difficulty identifying patients who benefit from resources to stay at work or return to work, either through MN RETAIN or other resources such as those for veterans, or for consideration of early referral to community workforce development programs such as American Job Centers, prompted a quality improvement project to determine a systematic method to capture employment status including whether a patient’s work was currently disrupted by a medical illness or injury.

Historic systems aimed to channel candidates to workforce development and work retention have not typically included the healthcare sector as an important stakeholder in early identification and referral of at-risk workers. Systematic collection of information on employment allows patients at risk of work disability to receive information about resources to support employment retention beginning with communication through the EMR combined with direct follow-up from healthcare staff to encourage use of available resources to reduce unemployment and increase workforce retention and productivity.

### Objectives

The objective of this continuous quality improvement project was to develop a systematic way to identify and contact patients who may benefit from services to help them stay at work or return to work when an injury or illness was acutely impacting work. The scope included identification of patients with newly diagnosed injuries or illnesses, those with exacerbations of chronic conditions acutely impacting work, and those with recent or upcoming surgeries or procedures impacting work. The goal of this quality improvement initiative was to ensure healthcare providers and teams were aware of their patient’s work status such that efforts could be made to prevent prolonged disruption of work and risk of occupational disability. The ability to reach patients whose work was impacted by an injury or illness—providing them with community and healthcare resources, such as free services through the MN RETAIN^12^ program, was evaluated as a one-use case example of the employment SDOH question.

## Materials and methods

**Figure 1.**
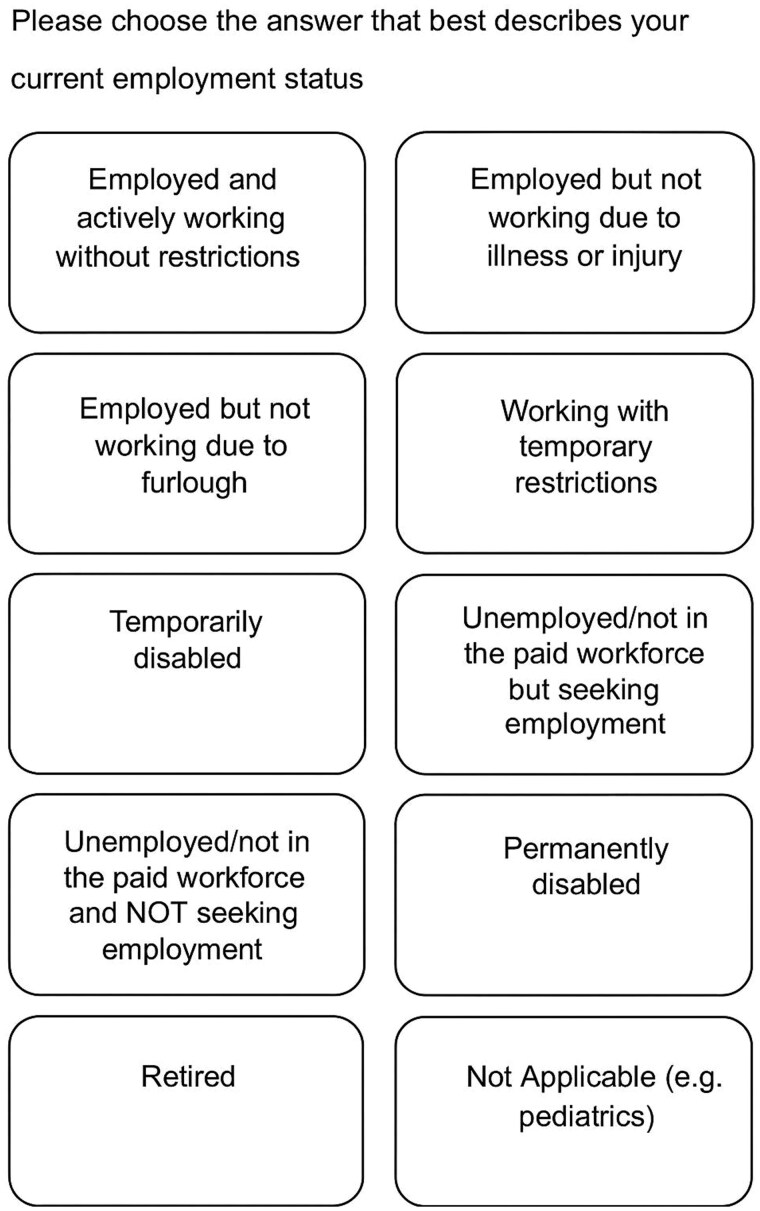
Employment as a social determinant of health question with response options.

This is a cohort study. Approval was obtained through the Patient Clinical Questionnaires and Surveys Oversight group to add a question focused on employment status to the institutional social determinants of health (SDOH) questionnaire within the EMR (Epic; Madison, Wisconsin) of a large multi-state medical center (Figure 1) that asked the patient to self-report the employment status that they felt best described their work ability. The employment question was added to the foundation set for SDOH and was combined with an existing question on financial resources. Other preexisting SDOH questions focused on transportation needs, housing stability, food insecurity, tobacco use, intimate partner violence, physical activity, alcohol and drug use, stress, social connections, and nutrition remained unchanged. The questionnaire was administered to all adult patients, ages 18 years and older, who sought care at any site within the healthcare system including clinics in the states of Minnesota, Arizona, Florida, and Wisconsin. While the question was added to the SDOH questionnaire for all patients, a focused intervention to provide information on resources to patients was developed for individuals living in MN. Patients were identified based on the state listed in their home address under demographics.

Ten response options identified through a subject matter expert group consisting of occupational medicine physicians and return to work professionals were identified and intended to cover all major categories of work status. The responses were then categorized based on the risk of new or acute work loss/disability with responses mapping to high risk (red), moderate risk (yellow), and low risk (green) (Figure S1). One of the primary factors impacting risk stratification was time since last work date with a focus on patients who have worked at least one day within the past three to six months. For patients who chose a question that indicated they were not working (temporarily disabled, furloughed, permanently disabled, employed but not working due to illness or injury, unemployed but looking for work), they were prompted to enter the last date they worked. Those patients who had worked more recently and had an injury or illness that was newly impacting their ability to work were felt to be at higher potential benefit for early interventions either through sharing community resources or receiving assistance from a healthcare return to work case manager. Patients at high risk of acute work disability included those who had worked at least one day in the past three to six months and were either employed but unable to work due to illness or injury or were temporarily disabled. The moderate-risk group consisted of individuals who were furloughed from their jobs, working with temporary restrictions, or unemployed but actively looking for work. Low-risk individuals were defined as those who were employed and working without restrictions, permanently disabled, retired, or unemployed and not seeking work. These classifications helped group patients for outreach based upon their potential for benefit from resources to help them stay at work or return to work.

A registry was then created within the EMR to include patients whose responses mapped to moderate or high risk of acute work disability, prompting outreach to patients via electronic or paper letters that provided descriptions of return-to-work (RTW) resources (Supplementary Material). The letters were available in English and also translated to the three most common languages for non-native English speakers identified within the patient population (Spanish, Somali, Arabic) for accessibility. The preference was to send the information electronically through the patient portal when possible as this did not add cost from mailing paper letters and also allowed for rapid communication of information to the patient. While the SDOH question was added to the single EMR used across all healthcare center sites, for Minnesota residents whose work had recently been impacted within the prior 6-month period and who were in the moderate- or high-risk categories for acute work disability, a dedicated campaign was initiated to reach those who may benefit from additional resources to help them stay at or return to work. One of the resources included was information about the MN RETAIN program, an initiative to provide early intervention services including free access to a return to work case manager focused on workforce retention to workers age 18 and older who live and work in MN and who have an injury or illness acutely impacting their work within the prior 6 months.^12^ Data for Minnesota residents who completed the SDOH questionnaire between February 1, 2023, and January 31, 2024, were collated. Individuals whose responses to the employment question mapped to high or moderate risk of acute work disability were included in an outreach campaign via the EMR. Patients with electronic portal access were sent an electronic message with information regarding resources that could be accessed including information about the MN RETAIN program. Patients who did not have access to the electronic portal to receive messages were sent a descriptive letter via US mail. A follow-up phone call to each patient potentially eligible for MN RETAIN^12^ was made after the portal messages (or paper letters for those who did not have electronic portal access) were sent. Those who were found to be eligible for the MN RETAIN program based on existing inclusion/exclusion criteria^12^ were offered enrollment in the study. The data presented here includes statistics on patients identified as being at moderate or high risk of work disability based upon the SDOH employment status question.

### Statistical analysis

Data analysis was performed using R software (version 4.4.2). Descriptive statistics were used to summarize patient demographics, status distributions, and temporal trends in reading status. Means and standard deviations (SD) were reported for continuous variables, while categorical variables were presented as frequencies and percentages. Comparisons across moderate and high-risk groups for acute work disability were conducted using appropriate statistical tests, including t-tests for continuous variables and chi-square tests for categorical variables, with a significance threshold set at *P* < .05. Geospatial analysis of patient distribution was conducted at the county level. Aggregated patient counts by county were merged with geographic data from the US counties map, specifically focusing on Minnesota. County names were standardized for compatibility, and choropleth maps were generated to visually represent patient counts using a gradient fill. Temporal analysis assessed the time taken to read the electronic EMR patient portal messages, grouped into intervals of ≤1 day, ≤7 days, and ≤30 days. Proportions were calculated for each category, and pie charts were constructed to visualize the relative proportions within each time interval. All figures were created using the *ggplot2* package, and the *finalfit* package was utilized to stratify and summarize data. Geospatial analysis of patient distribution was conducted at the county level using the *maps*, *mapdata*, and *sf* packages.

## Results

In total, 2059 patients were identified as having high or moderate risk of acute work disability; these groups were determined based on their answers to the SDOH employment question as displayed in Figure 1. Table 1 shows the characteristics of the participants including age, self-reported gender, and status of outreach. A minority of patients (102/2059, 5%) did not have access to the patient electronic portal, and the outreach messages were printed and mailed.

**Table 1.** Characteristics of participants stratified by risk.

Employment risk	High risk of acute work disability	Moderate risk of acute work disability	Total	*P*
Number of participants	1332 (64.7%)	727 (35.3%)	2059	
Age (years)				
Mean (SD)	43.4 (13.3)	35.7 (13.4)	40.7 (13.8)	<0.001
Sex				
Female	654 (49.1%)	371 (51.0%)	1025 (49.8%)	0.633
Male	677 (50.8%)	355 (48.8%)	1032 (50.1%)	
Nonbinary	1 (0.1%)	1 (0.1%)	2 (0.1%)	
Status of outreach communication				
Printed	68 (5.1%)	34 (4.7%)	102 (5.0%)	0.722
Read	1157 (86.9%)	628 (86.4%)	1785 (86.7%)	
Sent	107 (8.0%)	65 (8.9%)	172 (8.4%)	

**Figure 2.**
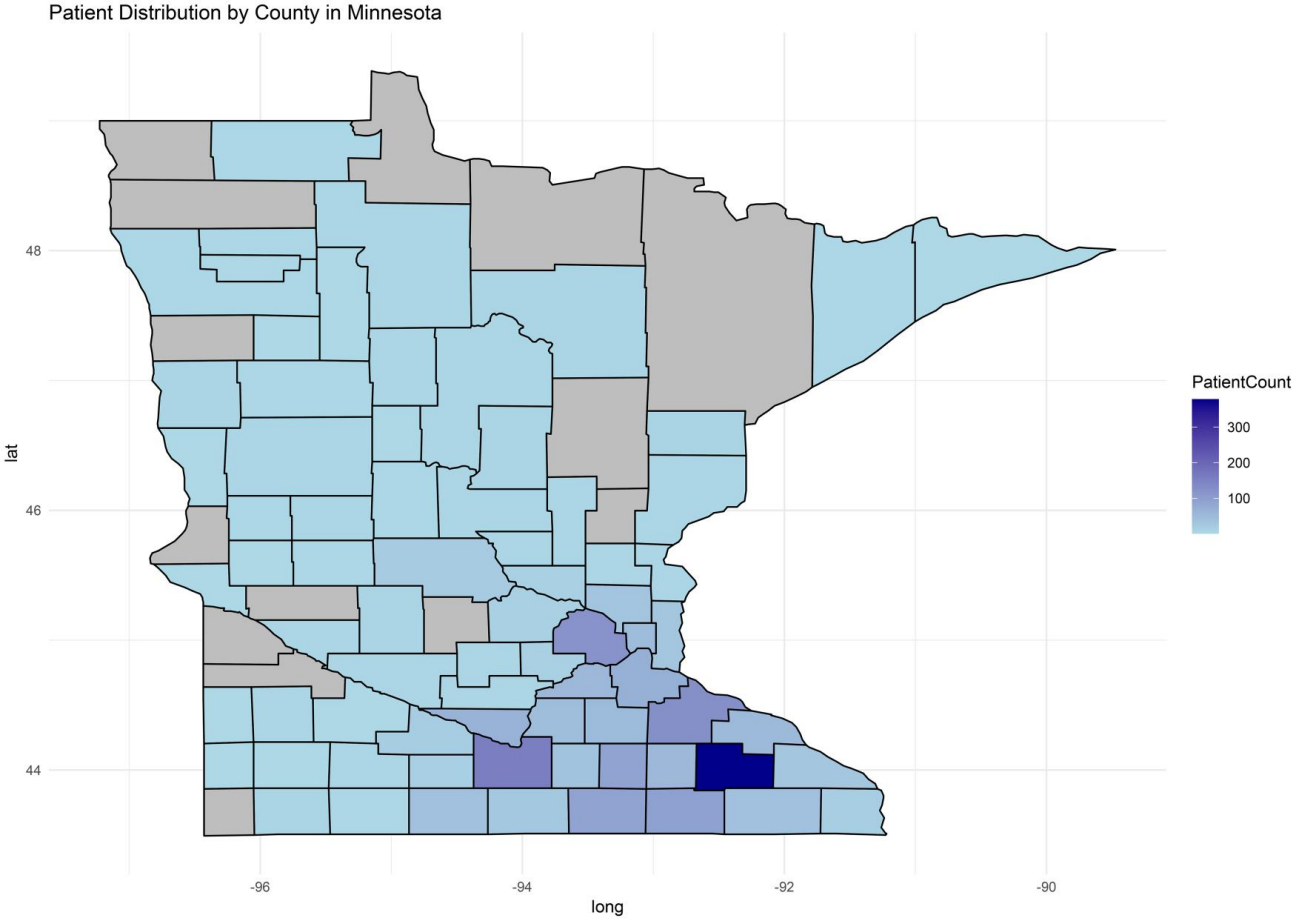
Patient distribution by counties in MN.

A total of 79 counties (90.8% of the total counties within the state) within Minnesota (MN) were represented among the 2059 patients who were identified as having high or moderate risk of acute work disability (Figure 2). Among the patients who received information electronically through the campaign via the patient portal (1957/2059; 95.05%), 86.7% read the message. The average time taken to read the message was 11.8 days (SD 22.6) for the subset of messages sent via the electronic patient portal (Table 2).

**Table 2.** Average number of days to read outreach portal messages by template language and format.

Outreach template^a^: *N* (%)	RETAIN initial outreach letters (English)	98 (4.8%)
	RETAIN Initial Outreach Letters (Spanish)	4 (0.2%)
	RETAIN Initial Outreach Messages (English)	1940 (94.2%)
	RETAIN Initial Outreach Messages (Arabic)	1 (0.0%)
	RETAIN Initial Outreach Messages (Somali)	2 (0.1%)
	RETAIN Initial Outreach Messages (Spanish)	14 (0.7%)
Status change to read (days) for outreach messages^b^: Mean (SD)		11.8 (22.6)

a “Outreach letters” refer to mailed paper letters, while “Outreach messages” refer to electronic messages sent through the patient portal.

b The “Mean (SD)” value refers to the average number of days between sending and status change to “read” for electronic portal messages only.

**Figure 3.**
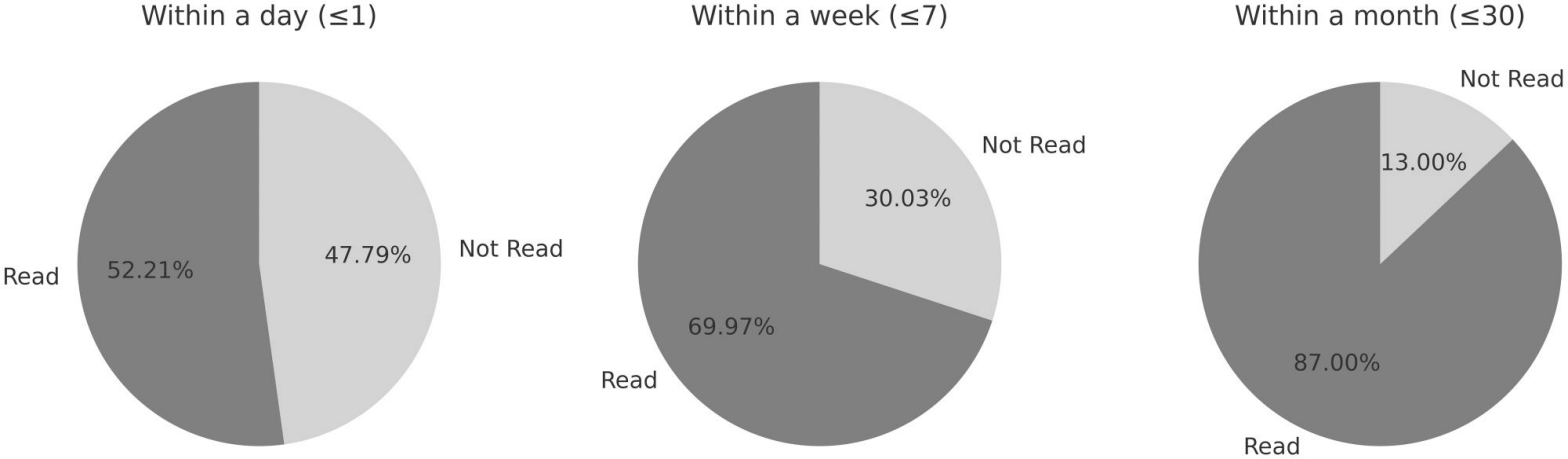
Percentage of patients reading the message over time.

Figure 3 shows that the patients’ engagement with the electronic EMR campaign messages increased over time. Of the 95% of patients who received the outreach message electronically, 52.21% read it within the first day and this number increased to 69.97% by the end of the first week. By the end of the month, 87.00% of patients had read the message, indicating that electronic communication reached most of the target population.

Phone outreach attempts by healthcare staff were also made to the 2059 MN patients who were identified as moderate or high risk of acute work disability. Typically, the first phone call was made within 1-3 business days of sending the outreach letter. Those who read the message within 1 week of receiving it would have seen the message prior to receiving the final phone outreach attempt from the healthcare team (a maximum of 3 total attempts were made for phone outreach). Based on individual phone calls which included screening for inclusion/exclusion criteria and assessment of the patient’s perceived benefit of engaging with a return-to-work case manager, patients were either offered enrollment in MN RETAIN or encouraged to access the other resources listed in the letter they received. One hundred and forty-four (144) patients (7% if the 2059 contacted) completed enrollment in the MN RETAIN study.

## Discussion

Employment status remains a significant social determinant of health and including information about ability to work allows for healthcare institutions to provide individualized resources targeted to specific patients who have a health condition impacting ability to work. In this analysis, the utility of adding self-reported employment status, including acute impact of injury or illness on work ability, allowed the healthcare team to identify patients who were eligible for a new program that many providers and patients would not have been aware of otherwise (MN RETAIN). Healthcare providers should recognize the importance of not only obtaining an occupational history but clearly establishing if a patient is employed and/or experiencing limitation in performing their occupation due to a health impairment. Preventing unnecessary work disability allows patients to maintain purposeful productive engagement and economic self-sufficiency—each vital to overall health and well-being. Within most healthcare systems, the care team does not have a way to identify which patients are at risk of acute work disability to be able to provide resources that may help patients maintain employment. Healthcare providers are in a unique position to assist patients who have not yet lost their jobs maintain their employment. A systematic change is needed to capture this important information regarding employment for every adult patient, just as information regarding alcohol and drug use, nutrition, sleep, and other social determinants of health are collected. Employment has many direct and indirect benefits to overall health. In the US, health insurance is often provided through employers, and separation from employment can impact access to needed healthcare services.

Li et al^13^ conducted a scoping review to explore SDOH data integration into EHRs with special interest on translational research and use of SDOH data to change healthcare. Of 324 studies included in the qualitative analysis, only 19 described an intervention within healthcare that was driven by the SDOH data in the EMR.^13^ Several studies within the analysis used geocoding data of addresses or other publicly available data to infer SDOH information for a population rather than capturing real-time individual results from a patient.^13^ While use of tools like natural language processing of free text data in clinical notes can capture a wealth of information, it is not often used on a recurring scheduled basis and has the limitation of unstructured data and multiple meanings of words within notes such as the term “work.” Chen et al^14^ conducted a systematic review that showed that integrating individual level SDOH into EMRs could assist with risk assessment as well as prediction of healthcare utilization and health outcomes.^14^ Of the 71 studies included in the final analysis, only 4% (3 studies) had the outcome of preventive health services, referral, or other intervention based on the SDOH data.^14^ The method described in this manuscript demonstrates the benefits of systematically identifying patients whose medical conditions are adversely impacting work ability and promptly reaching out to provide assistance.

Although only 7% (144/2056) of those identified as high to moderate risk of acute work disability chose to engage with the MN RETAIN program, without the addition of the employment question within the SDOH questionnaire, there would not have been a method to systematically identify and contact these patients to share resources. Due to the nature of the outreach, it is unclear how many of the additional patients may have taken action to access the other resources included in the letter provided such as services for veterans and through American Job Centers. However, the fact that over 87% of patients opened the message confirms that they received and were aware of the resources. The time to open the messages also varied significantly with the average time to read the message being 11.8 days. This indicates that some patients who have access to their patient portal may not be active users who promptly review messages received. Healthcare teams and clinicians expressed appreciation for the proactive approach to providing resources to patients who may be at risk of work disability based through this initiative. This systematic approach to getting resources to patients based on their SDOH response to the employment question was unique as the other SDOH questions required clinician intervention during the patient care visit. Work-related outcomes for the cohort of 144 patients who engaged with the MN RETAIN program are not yet available, however early data indicates that patients and their treating providers felt the inclusion of a return-to-work case manager who could serve as a resource for work-related concerns to be a benefit to all stakeholders.

A number of factors may have impacted the number of patients identified as being at risk of acute work disability as part of the outreach campaign. Within the healthcare institution where the question was implemented, the SDOH questionnaire is routinely administered on an annual basis per institutional practice to all patients based on the last date they completed the questionnaire as well as select interim times (ie, pre-surgical health screening). Increased frequency of administering the SDOH questionnaire would improve identification of patients who may have work impacted from their health at a different time of year (albeit this needs to be weighed against survey fatigue in some patients with chronic conditions). In general, we found that the patient’s self-identified work status accurately reflected their current employment. Although the outreach messages provided a mechanism to share resources with patients to support return to work, this was the first proactive initiative we are aware of where systematic outreach to patients was prompted by their response to the SDOH questionnaire. Since patients were not familiar with this type of patient portal messages and the message did not come from their treating provider, they may have been less motivated to open and read the message.

A uniform data model for SDOH components doesn’t exist within the U.S. Because of this, we are not able to aggregate data across healthcare systems or EHRs.^15^ While employment as a SDOH domain has evidence showing it is associated with health and useful to capture in healthcare it is not often included in the final domains within SDOH questionnaires.^16^^,^^17^ Since SDOH data is not collected in the same way across EHRs, an inherent barrier exists to implementing interventions to address the root SDOH domain of employment and to reduce risk of health effects stemming from unemployment. Our study provides one example of translating data collected into action with the hope that sharing this framework for capturing employment status may benefit other healthcare organizations and EHRs to incorporate this within their own systems. We agree with Kivimaki^8^ and colleagues that healthcare policy and practice changes addressing the social determinants of health could reduce health inequalities.^8^ Employment is a less frequently captured SDOH variable as it is not traditionally a domain that medical providers feel they can impact. However, with advances in accommodations of many disabilities and laws that require employers to consider such accommodations, awareness of health conditions adversely impacting work and prompt action to refer or support patients in remaining employed can have significant health effects.^18^

Finally, some patients who have electronic patient portal access may have forgotten their password or may not be regular users of the portal and therefore may not have promptly read the electronic message.

## Conclusion

Loss of employment has detrimental impacts on individual and population health. To enable medical providers to focus on the medical care needed for their patients to return to work, they first need to know which patients are at risk for loss of work. Patients who are employed but not working due to physical or mental impairments are at significant risk of work disability. By including employment as a SDOH in screening questionnaires captured in the EMR, this important information can be available for all adult patients seeking healthcare. Information about employment, work status (working or not), industry, and occupation are important, but have historically been absent from most electronic medical records. Healthcare delivery organizations and providers using the records should consider the importance of this information about patient employment, prompting further questioning on occupational and environmental exposures and to bolster both immediate pandemic recovery efforts and for the long-term health of communities. By automating these initial EMR patient portal messages, no additional burden was placed on the healthcare team but ensured that patients could receive information to support their employment prior to loss of their job. This manuscript describes a straightforward mechanism to capture information on employment in the electronic medical record using a minor modification to the SDOH questionnaire. By sharing this information, other healthcare institutions and EMRs may use this strategy to incorporate employment status into the SDOH within their system as one way to support the workforce.

## Supplementary Material

ooaf108_Supplementary_Data

## Data Availability

Data analyzed during this study is available from the corresponding author on reasonable request.

## References

[1] Ramar K , Malhotra RK, Carden KA, et al Sleep is essential to health: an American academy of sleep medicine position statement. J Clin Sleep Med. 2021;17:2115-2119. 10.5664/jcsm.9476 34170250 PMC8494094

[2] Gehlich KH , Beller J, Lange-Asschenfeldt B, Köcher W, Meinke MC, Lademann J. Consumption of fruits and vegetables: improved physical health, mental health, physical functioning and cognitive health in older adults from 11 European countries. Aging Ment Health. 2020;24:634-641. 10.1080/13607863.2019.1571011 [published Online First: 20190207]. 30729805

[3] Volpp KG , Mohta NS. Patient engagement survey: social networks to improve patient health. NEJM Catalyst. 2018;4. https://catalyst.nejm.org/doi/pdf/10.1056/CAT.18.0285

[4] O’Connor DB , Thayer JF, Vedhara K. Stress and health: a review of psychobiological processes. Annu Rev Psychol. 2021;72:663-688. 10.1146/annurev-psych-062520-122331 32886587

[5] Health WCoSDo, Organization WH. Closing the Gap in a Generation: Health Equity through Action on the Social Determinants of Health: Commission on Social Determinants of Health Final Report. World Health Organization, 2008.

[6] Waddell G , Burton AK. *Is Work Good For Your Health and Well-Being?* London: The stationary office; 2006. https://assets.publishing.service.gov.uk/media/5a7cd68640f0b6629523c1de/hwwb-is-work-good-for-you-exec-summ.pdf

[7] Crayne MP. The traumatic impact of job loss and job search in the aftermath of COVID-19. Psychol Trauma. 2020;12:S180-S182. 32478539 10.1037/tra0000852

[8] Kivimäki M , Batty GD, Pentti J, et al Association between socioeconomic status and the development of mental and physical health conditions in adulthood: a multi-cohort study. Lancet Public Health. 2020;5:e140-e49. 32007134 10.1016/S2468-2667(19)30248-8

[9] Cash R , Patel V. Has COVID-19 subverted global health? Lancet. 2020;395:1687-1688. 32539939 10.1016/S0140-6736(20)31089-8 PMC7200122

[10] Woolf SH , Chapman DA, Sabo RT, Weinberger DM, Hill L. Excess deaths from COVID-19 and other causes, March-April 2020. JAMA. 2020;324:510-513. 32609307 10.1001/jama.2020.11787 PMC7330820

[11] Statistics UBoL. Effects of COVID-19 Pandemic on the Employment Situation. Secondary Effects of COVID-19 Pandemic on the Employment Situation. 2021. https://www.bls.gov/news.release/archives/empsit_05072021.htm

[12] Breeher L , Omondi N, Westphal S, et al Mn RETAIN: an early intervention model to identify and support stay at work and return to work for injured and ill employees across industries. Work. 2025;80:1415-1424. 40297873 10.1177/10519815241290331

[13] Li C , Mowery DL, Ma X, et al Realizing the potential of social determinants data in EHR systems: a scoping review of approaches for screening, linkage, extraction, analysis, and interventions. J Clin Transl Sci. 2024;8:e147. 10.1017/cts.2024.571 39478779 PMC11523026

[14] Chen M , Tan X, Padman R. Social determinants of health in electronic health records and their impact on analysis and risk prediction: a systematic review. J Am Med Inform Assoc. 2020;27:1764-1773. 10.1093/jamia/ocaa143 33202021 PMC7671639

[15] Cantor MN , Thorpe L. Integrating data on social determinants of health into electronic health records. Health Aff (Millwood). 2018;37:585-590. 10.1377/hlthaff.2017.1252 29608369 PMC10995852

[16] Io M. Capturing Social and Behavioral Domains in Electronic Health Records: Phase 1. The National Academies Press, 2014. 24757748

[17] Io M. Capturing Social and Behavioral Domains and Measures in Electronic Health Records: Phase 2. The National Academies Press, 2014. 25590118

[18] Division UDoJaCR. Americans with Disabilities Act. Secondary Americans with Disabilities Act. The Americans with Disabilities Act | ADA.gov.

