# Supplementary material for: Adding employment status in the medical record demonstrates its importance as a social determinant of health
Source: JAMIA Open. 2025 Oct 1;8(5):ooaf108. doi: 10.1093/jamiaopen/ooaf108 (PMC12488229; doi:10.1093/jamiaopen/ooaf108)
Supplement: ooaf108_Supplementary_Data [file ooaf108_supplementary_data.zip › MNResidentsLetter_FINAL.docx]

Dear [Patient Name],

Thank you for choosing us for your healthcare needs. Our mission is to inspire hope and contribute to health and well-being by providing the best care to every patient through integrated clinical practice, education, and research. Our goal is to provide you with comprehensive health care services to meet your needs.

At your recent visit with one of our providers, you completed a form that asked about concerns and needs you may have, including food, housing, safety, and work. Based on your answers, we identified that you may be interested in services to help you with work or your career; for example, finding a new job or receiving services to help you stay in your job.

We want to share information with you about Minnesota’s **R**etaining **E**mployment and **T**alent after **I**njury/**I**llness **N**etwork (RETAIN) and other job-related services.

Due to living in Minnesota, you may qualify for free **RETAIN services**. RETAIN is a grant program funded by the U.S. Department of Labor’s Office of Disability Employment Policy (DOL/ODEP) in partnership with the Employment and Training Administration and the Social Security Administration. The Minnesota RETAIN team has a primary goal of providing early intervention strategies to help people stay at or return to work after an injury or illness.

RETAIN may help injured and ill workers:

- Maintain an employment relationship that provides job security
- Protect their self-esteem
- Cultivate their job skills
- Maximize their physical health and rehabilitation
- Resume life and relationships at work

To learn more about RETAIN, contact a RETAIN team member at:

RETAIN

Phone: 507-284-4537

Email: [RETAIN@mayo.edu](mailto:RETAIN@mayo.edu)

The following are additional resources that may be of interest to you:

- **American Job Centers:**

<https://www.careeronestop.org/LocalHelp/AmericanJobCenters/american-job-centers.aspx>

American Job Centers provide free help to job seekers for a variety of career and employment-related needs. Nearly 2,400 American Job Centers, funded by the U.S. Department of Labor’s Employment and Training Administration, are located throughout the United States.

American Job Centers can help you look for work and offer job search workshops, free computer access, and more. To find out the closest American Job Center to your location, enter your city, state, or zip code on the following web page where it says “Location” under “Find an American Job Center.”

- **CareerOneStop**

[www.careeronestop.org](http://www.careeronestop.org)

The U.S. Department of Labor’s Employment and Training Administration toll-free help line helps job seekers connect with career counseling, job placement, job readiness, and life skills development offered by American Job Centers located across the country. Information is available in over 140 languages. Text Telephone capabilities, also called TTY, are available for the persons who are hearing impaired, by calling 1-877-889-5627. Call 1-877-US-2JOBS (1-877-872-5627) for assistance.

- **Job Accommodations Network**

<https://askjan.org/>

The Job Accommodation Network (JAN) provides free, expert, and confidential guidance on workplace accommodations and disability employment issues. JAN provides free consulting services for all employees, regardless of your medical condition. Services include one-on-one consultation about all aspects of job accommodations. You can contact JAN and request consultation or information services by the following methods: JAN’s office hours are 9 am to 6 pm Eastern, Monday through Friday by calling 1-800-526-7234, TTY at 1-877-781-9403, or via text to 304-216-8189.

Thank you for trusting us with your care. If you have any questions about these or other resources, please do not hesitate to contact the MN RETAIN team.

Sincerely,

Signature line MN RETAIN
