# Supplementary material for: Adding employment status in the medical record demonstrates its importance as a social determinant of health
Source: JAMIA Open. 2025 Oct 1;8(5):ooaf108. doi: 10.1093/jamiaopen/ooaf108 (PMC12488229; doi:10.1093/jamiaopen/ooaf108)
Supplement: ooaf108_Supplementary_Data [file ooaf108_supplementary_data.zip › Supplementary_figure.docx]

Supplementary figure:


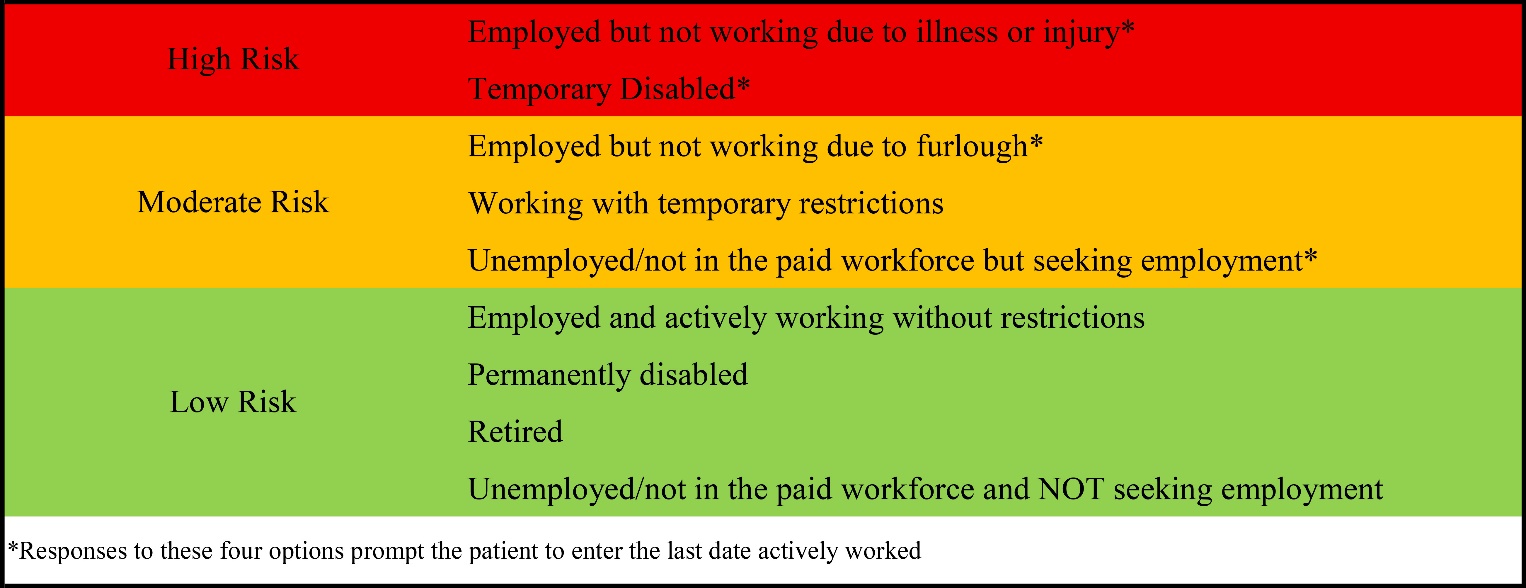


**Supplementary Figure 1: Employment SDOH response by acute work disability risk category**
